# Atomic Layer Deposition of Superconductive Niobium Carbonitride Thin Films

**DOI:** 10.1021/acs.chemmater.5c01456

**Published:** 2025-08-19

**Authors:** Paloma Ruiz Kärkkäinen, Anton Vihervaara, Timo Hatanpää, Katja Kohopää, Mikko J. Heikkilä, Marco Marín-Suárez, Kestutis Grigoras, Kenichiro Mizohata, Georgi Popov, Mykhailo Chundak, Antti Kemppinen, Matti Putkonen, Mikko Ritala

**Affiliations:** † Department of Chemistry, 3835 University of Helsinki, FI-00014 Helsinki, Finland; ‡ VTT Technical Research Centre of Finland Ltd, QTF Centre of Excellence, P.O. Box 1000, FI-02044 Espoo, Finland; § Department of Microelectronics and Quantum Technology, VTT Technical Research Centre of Finland, P.O. Box 1000, FI-02044 Espoo, Finland

## Abstract

Transition metal carbonitrides (TMCN) are stable materials with excellent catalytic and superconductive properties. Atomic layer deposition (ALD) stands out as the optimal method for the fabrication of these materials, enabling their use in future applications. In this study, we deposit ALD NbC_*x*_N_*y*_ films at 250–450 °C with NbF_5_ and 1,4-bis­(trimethylsilyl)–1,4-dihydropyrazine on Si, Ru, TiN, and soda lime glass. We analyze the film growth characteristics, composition, and phase. The films show substrate-enhanced growth on Si with a growth per cycle (GPC) of 1.3 Å. Additionally, the films were superconductive as-deposited and had a superconducting critical temperature (*T*_*c*_) of 14.5 K after annealing at 950 °C. This work expands the range of TMCNs deposited by ALD and demonstrates the applicability of ALD for thin film materials with a high *T*_*c*_.

## Graphical abstract

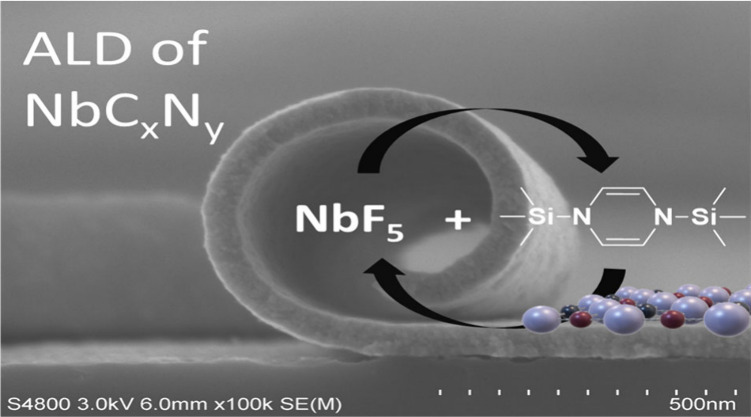

## Introduction

Transition metal carbides (TMC) and nitrides (TMN) are hard and strong materials with good chemical inertness and high melting points. They have conductivities close to the respective metals, their catalytic properties rival those of noble metals, and many of them exhibit superconductivity. These properties result from the unique structures of TMCs and TMNs, where the metal–carbon and metal–nitrogen bonds exhibit a combination of covalent, ionic, and metallic characteristics.^1,2^

Transition metal carbonitrides (TMCNs) are solid solutions of TMCs and TMNs. By developing new TMCNs, we can leverage properties of both carbides and nitrides, and in some cases even exceed their capabilities.^3^ For example, the critical temperature for superconductivity (*T*_*c*_) is 11.1 K for NbC,^4^ 16.0 K for NbN,^5^ and 17.8 K for NbCN.^6^ Furthermore, the carbon to nitrogen ratio (C/N ratio) can often be tuned by adjusting the film deposition parameters, enabling tailored properties to meet specific applications. For example, work function^7,8^ and *T*_*c*_ ^9^ have been tuned by varying the C/N ratio of TMCN films.

The capabilities of TMCN thin films have been demonstrated through studies on various applications in catalysis,^10–12^ wear resistance,^13–15^ and semiconductor devices.^7,16–18^ However, one of the most promising applications for TMCN films lies in quantum technologies. Both NbC and NbN exhibit superconductivity and have been widely studied.^19,20^ Their high *T*_*c*_ is practical for several applications as it reduces the requirements for cooling devices to very low temperatures. In addition, the critical current density of Josephson junctions according to the Ambegaokar-Baratoff relation is *J*_*c*_ = πΔ/(2*eR*_*T*_ *A*) where Δ ≈ 1.764*k*_*B*_ *T*_*c*_ is the superconducting energy gap (*k*_*B*_ is the Boltzmann constant) of the electrodes of the junction, *e* is the elementary charge, and *R*_*T*_ and *A* are the tunneling resistance and the area of the junction, respectively. High *T*_*c*_ thus allows to increase the critical current density compared to niobium (*T*_*c*_ = 9.2 K)^21^ and other single-element materials, and allow, e.g., superconducting quantum bits at elevated temperatures.^22,23^ Due to their high normal state resistivity, thin TMCN films exhibit high sheet kinetic inductance, *L*_□_ = *hR*_□_/(2π^2^ Δ), where *h* is the Planck constant and *R*_□_ is the sheet resistance of the film. High *L*_□_ enables, e.g., superconducting nanowire single-photon detectors (SNSPDs),^24,25^ and yields a promising direction for miniaturizing quantum computers.^26,27^ Recently, atomic layer deposited (ALD) TaC_*x*_N_1–*x*_ was demonstrated as a promising candidate for superconducting quantum circuits.^28^

For the various applications listed above, it is vital to be able to deposit thin films with methods that offer exact control over the film growth. ALD is the only deposition method where the film growth is self-limiting, allowing for an exceptionally precise control of film thickness and uniformity on complex three-dimensional structures.^29^ Although ALD of TMCN films is still in its early stages, WC_*x*_N_*y*_,^7,16,30–32^ TaC_*x*_N_*y*_,^28,33^ MoC_*x*_N_*y*_,^18,34,35^ and TiC_*x*_N_*y*_ ^8^ have already been deposited. Only one of these processes, WC_*x*_N_*y*_,^30^ does not use plasma-enhancement. Although plasma-enhanced ALD (PEALD) is frequently used because it enables low deposition temperatures and high deposition rates, thermal ALD is preferred, since the aggressive nature of the plasma species can be detrimental to the underlying layers and the recombination of plasma species limits the conformality of the films.

1,4-Bis­(trimethylsilyl)–1,4-dihydropyrazine, (Me_3_Si)_2_DHP and its analogue, (Me_3_Ge)_2_DHP, have been demonstrated as effective reducing agents for metal chloride precursors in the ALD of Pd,^36^ MoC_*x*_,^37,38^ Au,^39^ Ni,^40^ Ti,^41^ and Sn.^42^ Reaction mechanism studies show that the reducing agents react with the metal chloride terminated surface, M-Cl_*x*_, and form volatile Me_3_Si–Cl and Me_3_Ge–Cl as byproducts.^36,41^ The presumably antiaromatic 1,4-dihydropyrazine intermediate is subsequently oxidized into volatile aromatic pyrazine, thereby reducing the metal ions on the surface. Of the published processes, only those with Mo result in nonelemental films with (Me_3_Si)_2_DHP or (Me_3_Ge)_2_DHP. We recently proposed that the cause for the MoC_*x*_ deposition instead of Mo is the reaction of the pyrazine with the reduced Mo metal atoms during the deposition. Due to the chemical and physical similarities of Mo and Nb, we aimed to deposit NbC_*x*_ with a Nb halide and (Me_3_Si)_2_DHP. Since MoCl_5_ showed etching during the MoC_*x*_ depositions, and NbCl_5_ is known for its even stronger etching capability,^43,44^ we turned to NbF_5_ instead of NbCl_5_. Literature indicates that NbF_5_ would not etch Nb films in the same manner as NbCl_5_.^45–47^ We expect metal fluorides to react with the dihydropyrazine precursors (Me_3_Si)_2_DHP and (Me_3_Ge)_2_DHP similarly to metal chlorides. Fluorine has higher affinity than chlorine toward the Me_3_Si and Me_3_Ge groups of the dihydropyrazine precursors,^48^ but on the other hand fluorine is usually more strongly bonded also to metals.^49^ The ALD reaction mechanisms for the reduction of SnCl_4_ to Sn with (Me_3_Si)_2_DHP^42^ and AuClP­(CH_2_CH_3_)_3_ to Au with (Me_3_Ge)_2_DHP^39^ have been studied, and based on those studies the reduction of NbF_5_ is expected to proceed as shown in Figure 1 (metal route).

**Figure 1:**
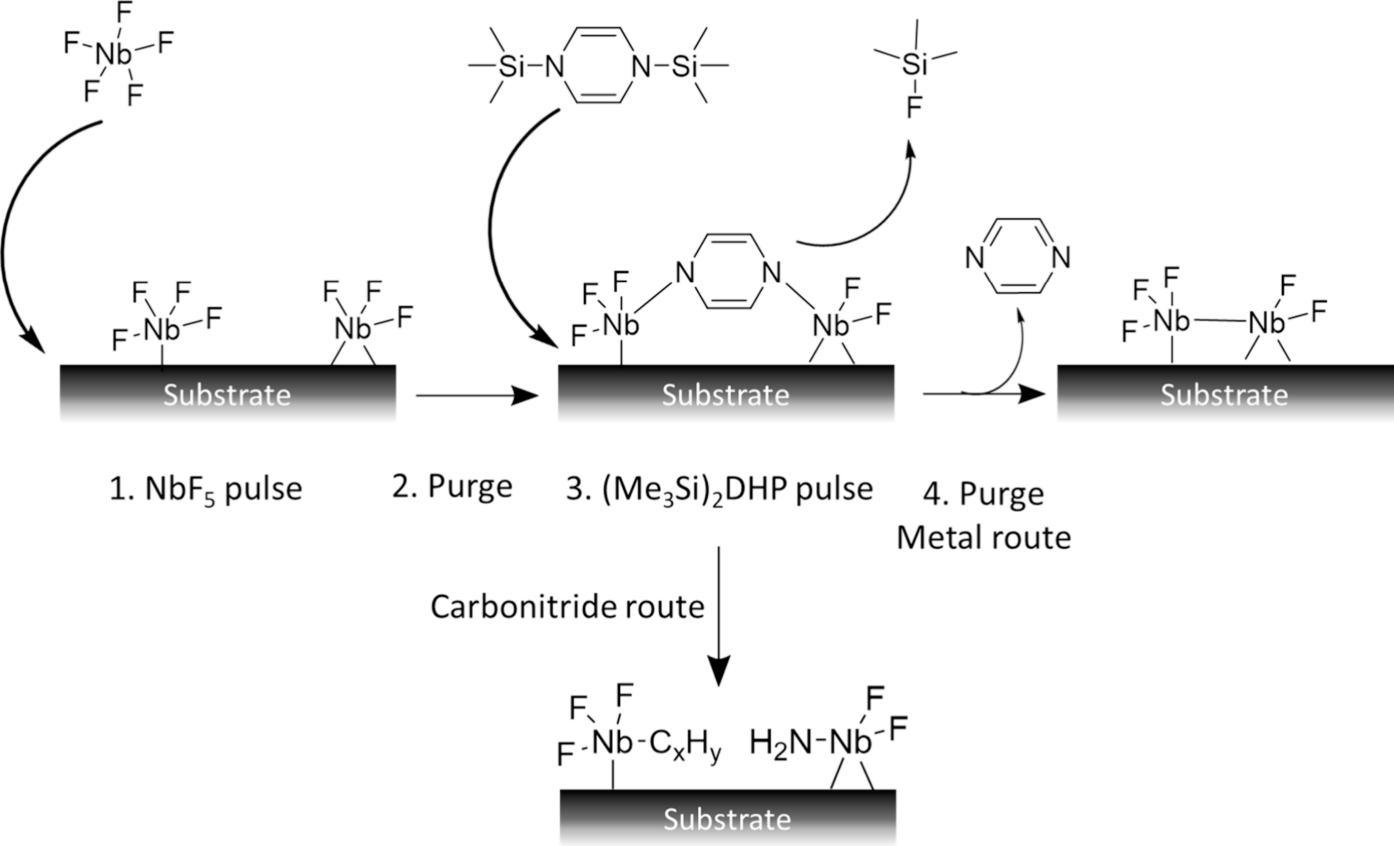
Suggested mechanisms for the deposition of NbC_*x*_N_*y*_ films with NbF_5_ and (Me_3_Si)_2_DHP at 425 °C.

However, because the resulting films are NbC_*x*_N_*y*_ instead of metallic niobium, it appears that, similar to the ALD process of MoC_*x*_, and contrary to the schematic in Figure 1 (metal route), the pyrazine ring reacts with niobium and incorporates nitrogen and carbon into the films (carbonitride route). The exact mechanism for this requires further studies. The N_2_ carrier gas used in the reactor is not likely the source of nitrogen in the films, as has been confirmed previously in our studies with MoCl_5_ and (Me_3_Ge)_2_DHP in the ALD of MoC_*x*_.^37^

In this study, we present a novel thermal ALD process for NbC_*x*_N_*y*_. We deposited the films with NbF_5_ and (Me_3_Si)_2_DHP at 250–450 °C, (Me_3_Si)_2_DHP acting as a reducing agent, and carbon and nitrogen precursor. This means that We studied the ALD growth properties, film crystallinity and composition as well as resistivity and superconducting critical temperature.

## Experimental Section

### Thermal Analysis

The thermal decomposition of (Me_3_Si)_2_DHP was studied using a Mettler Toledo TGA/DSC 3+ with a single differential thermal analysis (SDTA) sample holder.

### Film Deposition

The films were deposited by ALD in an ASM F-120 cross-flow, hot-wall reactor with a purifier (SAES Pure Gas, MC1–902F, H_2_O, O_2_, CO, CO_2_, H_2_, nonmethane hydrocarbons removal <1 ppb) for the carrier gas (N_2_, Linde 99.999%). During the depositions, the pressure inside the reactor was 5–10 mbar. The sublimation temperatures for the precursors were 32 and 35 °C for NbF_5_ (abcr, 99%) and (Me_3_Si)_2_DHP, respectively. (Me_3_Si)_2_DHP was synthesized in-house using the literature method by Sulzbach et al.^50^ In the synthesis, granular lithium was used instead of lithium powder, and yields similar to that reported^50^ were achieved.

Both precursors were loaded into the reactor by a method described previously^37^ due to their high air sensitivity. The precursors did not come in contact with ambient air at any point of the loading and remained under a N_2_ environment. The films were deposited on 5 × 5 cm^2^ Si (native oxide), soda lime glass, and on Ru and TiN films deposited on silicon. The Ru substrate consisted of a Si substrate covered by 10 nm of ALD Ru. The TiN substrate consisted of a Si substrate with 50 nm of ALD SiO_2_, covered by 2 nm of ALD TiN.

### Film Characterization

We determine the film thickness by performing Energy-dispersive X-ray spectroscopy (EDS) with an Oxford INCA 350 spectrometer connected to a Hitachi S-4800 scanning electron microscope. The nominal film thicknesses were calculated from Nb L X-rays with a GMRFILM program^51^ using film densities obtained by X-ray reflectivity (XRR, Rigaku Smartlab). Film morphologies were studied with Field Emission Scanning Electron Microscopy (FESEM, Hitachi S-4800). X-ray diffraction (XRD) measurements on film crystallinity were performed with a Rigaku Smartlab instrument using parallel beam optics and Cu Kα (λ = 1.54 Å) radiation at an incident angle of 1°. The data analysis was performed with a PANalytical Highscore Plus 4.1 software. The elemental film compositions were measured with time-of-flight elastic recoil analysis (ToF-ERDA) with 5MV tandem accelerator at the University of Helsinki. In the measurements, an ion beam of 30 MeV 127I^+7^ was used. The detector was at a 40° angle and the angle of the incident ion beam was 16 °C relative to the sample surface.

X-ray photoelectron spectroscopy (XPS) measurements were performed to determine the chemical states of the elements in the films. The instrument consisted of a PREVAC company system equipped with an EA-15 hemispherical electrostatic energy analyzer and an RMC50 monochromatic X-ray source with Al Kα anode (1486.7 eV). The analyzer pass energy was 100 eV and the slit size was 0.8 × 25 mm^2^. The pressure during the measurements was 10^–10^ mbar. Sputtering was performed with 2 keV Ar^+^ ions with a 5 mA emission current over an area of 5 × 2 mm^2^ for 60 min. XPS peak fitting was carried out with CasaXPS software (V 2.3.26PR1.0). The Nb 3d_5/2_ and 3d_3/2_ spectra were fitted with the spin–orbit splitting set to 2.72 eV.

A confocal Raman microscope (NT-MDT Ntegra) with a 532 nm laser and a 100× objective was used to measure micro-Raman spectra in backscattering geometry. The incident laser power was 4.5 mW. The measurements were done with 500 exposures that were 0.2 s long. The sheet resistances of the films were measured at room temperature with a four-point probe (CPS Probe Station, Cascade Microtech, combined with a Keithley 2400 SourceMeter). The sheet resistances were measured from films deposited on poorly conductive Si (n-type, slightly doped), and resistivities were calculated by multiplying the sheet resistances with film thicknesses.

### Annealing

We annealed several films in a Jipelec RTP tool. The sample pieces to be annealed were put on a 150 mm silicon wafer carrier which was laying on three quartz pins inside the annealing chamber. A thermocouple calibrated pyrometer was used for the temperature control. After loading the samples, the chamber was evacuated until the pressure was below 1 × 10^–2^ mbar. Then, a nitrogen gas flow was activated and kept at ∼ 10 mbar pressure inside the chamber during the whole annealing process. Annealing consisted of four steps: about 20 s of ramp-up, 300 s at the fixed temperature (650, 750, 850, or 950 °C), about 20 s of ramp-down to ∼ 400 °C, and about 10 min cool-down to ∼ 150 °C. The sample was exposed to ambient air after opening the lid of the chamber. To evaluate the influence of nitrogen gas during annealing, some tests were performed in vacuum, without gas flow. In this case, the pressure inside the chamber was kept below 1 × 10^–2^ mbar during the whole annealing process.

To minimize possible oxidation, the annealed and reference (as-deposited) films were stored in a nitrogen cabinet until the subsequent measurements and tests.

### Cryogenic Characterization

The critical temperature (*T*_*c*_) of superconductivity was measured by cooling down the films using a pulse tube cryostat with a base temperature of about 3.5 K. Measurements were performed on small, cleaved pieces that were wire-bonded to a sample holder with four wires to allow resistance measurement. The sample holder was connected to sets of resistive coaxial or twisted-pair loom cables, which electrically connect the low temperature stages of the refrigerator to room-temperature connectors. At room temperature, we used a lock-in amplifier sending small excitation currents of 1 μA through two wire bonds and detecting the voltage drop between the remaining ones. The uncertainty in the geometrical configuration of the wire bonds only allows to measure relative resistance, and therefore the curves showing the superconducting transitions are shown with arbitrary units. We define the value of *T*_*c*_ as the temperature were resistance drops to half of the normal-state value at low temperature (*R*(*T*_*c*_) = 0.5 × *R*(20K)).

## Results and Discussion

### Thermal Stability of (Me_3_Si)_2_DHP

The thermal stability of the (Me_3_Si)_2_DHP reducing agent was examined because the deposition temperatures used in this study are significantly higher than those in previous ALD processes (170–385 °C).^38,41,42^ A sample of (Me_3_Si)_2_DHP was sealed into a high-pressure stainless-steel pan. Aluminum pans were not applicable as the pressure building up during the heating of the sample ruptured the pans causing fast evaporation of the sample before any decomposition related events were seen in the SDTA curve. A (Me_3_Si)_2_DHP sample heated from 25 to 500 °C with a rate of 10 °C/min showed an endotherm due to melting and then nothing until ∼ 370 °C, where an exothermic change, believed to be due to the thermal decomposition, began (Figure 2). At 480 °C the pan started to leak and the sample and decomposition products burst out. This measurement gives an estimate of the thermal stability of the (Me_3_Si)_2_DHP, though the result may not fully be representative of the ALD conditions.

**Figure 2:**
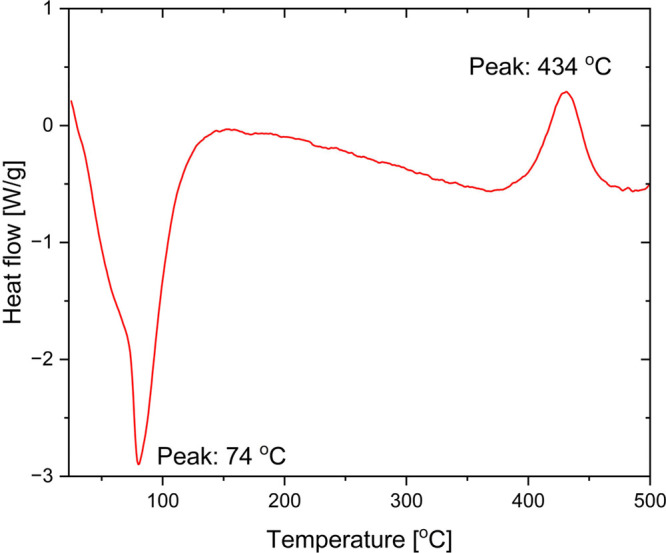
SDTA curve measured for (Me_3_Si)_2_DHP using a hermetically sealed high-pressure stainless-steel pan. A heating rate of 10 °C/min was applied.

### Film Deposition

We deposited NbC_*x*_N_*y*_ thin films with NbF_5_ and (Me_3_Si)_2_DHP at 250–450 °C. All the films were visually metallic (Figure 3). We studied the atomic compositions of the films by ToF-ERDA (Figure 3b). Interestingly, the niobium, nitrogen, and oxygen contents remained the same (∼28, ∼13, and ∼7 at. %, respectively) at all the deposition temperatures while the carbon content increased from 26 to 42 at. % in the films deposited above 350 °C (Figure 3b). A clear drop of fluorine content from 12 to 3 at. % was also observed with increasing deposition temperature. The resistivities of the films deposited at different temperatures were also measured. The lowest values of 140–170 μΩcm were obtained for the films deposited at 375–425 °C (Figure 3c). We decided to study the ALD growth behavior of this process at 425 °C based on the low resistivity and fluorine content of the films deposited at this temperature. The film composition will be discussed in more detail in Section Composition and Phase Analysis.

**Figure 3:**
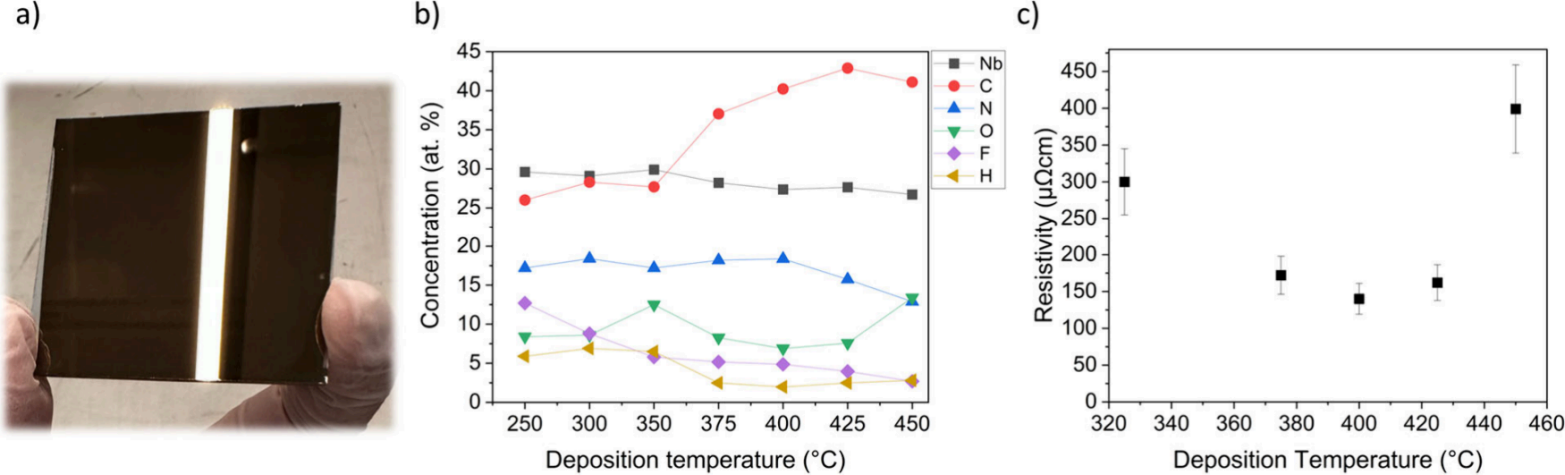
Photograph of a NbC_*x*_N_*y*_ film deposited on glass at 425 °C (a). The white stripe is cast on the film from the lighting fixture above it. Elemental composition of NbC_*x*_N_*y*_ films deposited at different temperatures (b). Resistivities of NbC_*x*_N_*y*_ films deposited at different temperatures (c).

We studied the saturation of the process on Si substrates by varying the pulse and purge lengths of both precursors (Figure 4). The growth per cycle (GPC) saturates at precursor pulses of 2.0 s for NbF_5_ and 3.0 s for (Me_3_Si)_2_DHP. Doubling the purge lengths from 1.0. to 2.0 s had no effect on the GPC. The purge lengths were therefore kept at 1.0 s for all the subsequent depositions. The saturated GPC is 1.3 Å on Si and 0.8 Å on TiN and Ru. The density of the films deposited at 425 °C on Si was 4.8 g/cm^3^ as measured with XRR. We did not observe any film etching by NbF_5_ during the depositions.

**Figure 4:**
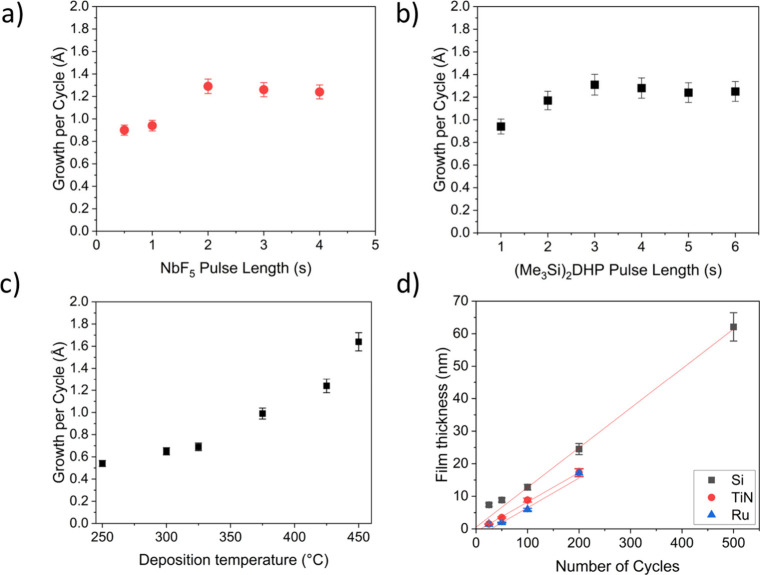
NbC_*x*_N_*y*_ film GPCs as a function of NbF_5_ (a) and SiDHP (b) pulse lengths and deposition temperature. NbC_*x*_N_*y*_ film GPC as a function of deposition temperature on Si substrates (c). NbC_*x*_N_*y*_ film thicknesses as a function of the number of ALD cycles on Si, TiN, and Ru substrates at 425 °C (d).

We studied the growth of the films in more detail on Si, TiN, and Ru substrates. The growth is linear as a function of the number of ALD cycles on all substrates (Figure 4d) but seems to have substrate-enhanced growth on Si. We further investigated the substrate-enhanced growth behavior of NbC_*x*_N_*y*_ on Si by giving only one 3.0 s pulse of NbF_5_ on Si at 425 °C and studying the surface with SEM, EDS, XRD, and XPS. The SEM and XPS results show that during the first cycle, NbF_5_ reacts with the native oxide terminated silicon substrate surface, producing scattered islands. During the next few cycles, similar reactions may repeat, until the film is continuous and the substrate is no longer exposed. These reactions are likely the cause of the substrate-enhanced film growth on Si substrates. The composition of the islands could not be unambiguously determined during this study. Further details on the measurements can be found in the Supporting Information.

To study film continuity, we deposited films on all substrates with 25, 50, 100, and 200 ALD cycles. SEM images show the films to be continuous already after 25 cycles on Si and after 50 cycles on TiN and Ru (Figure S3., Figure 5). The film is smooth on Si after 25 cycles (7.4 nm in thickness), but bubble-like structures seem to form after 50 cycles (8.9 nm) and become more prominent as the cycle number increases. The small difference in film thicknesses on Si after 25 and 50 cycles can be attributed to the substrate enhanced growth during the initial growth stage, as can be seen from Figure 4d. A cross-sectional image reveals the films to have slightly blistered from the Si substrate (Figure 6). This behavior is not seen on the Ru substrate after 100 cycles (Figure 6). The blisters likely form during the cooling of the films after the deposition due to a significant difference in the coefficient of thermal expansion (CTE) between the substrates and the film (Table 1). In lieu of a representative reference for NbC_*x*_N_*y*_, we used the CTE of NbC. The CTE of Ru is the closest to NbC, which could explain why the films appear uniform on Ru but not on Si or TiN. The CTEs of Si and SiO_2_ are much lower than that of NbC, which means that the substrate contracts much less than the film during cooling, causing stress in the film. This leads to the delamination of the film off the substrate. The higher amount of blisters in the thicker films on Si support this conclusion: the thicker the film, the higher the stress forming during cooling.

**Figure 5:**
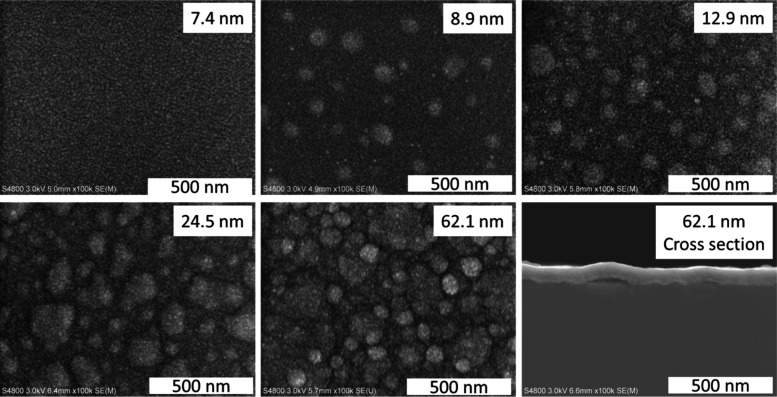
SEM images of NbC_*x*_N_*y*_ films deposited on Si at 425 °C.

**Figure 6:**
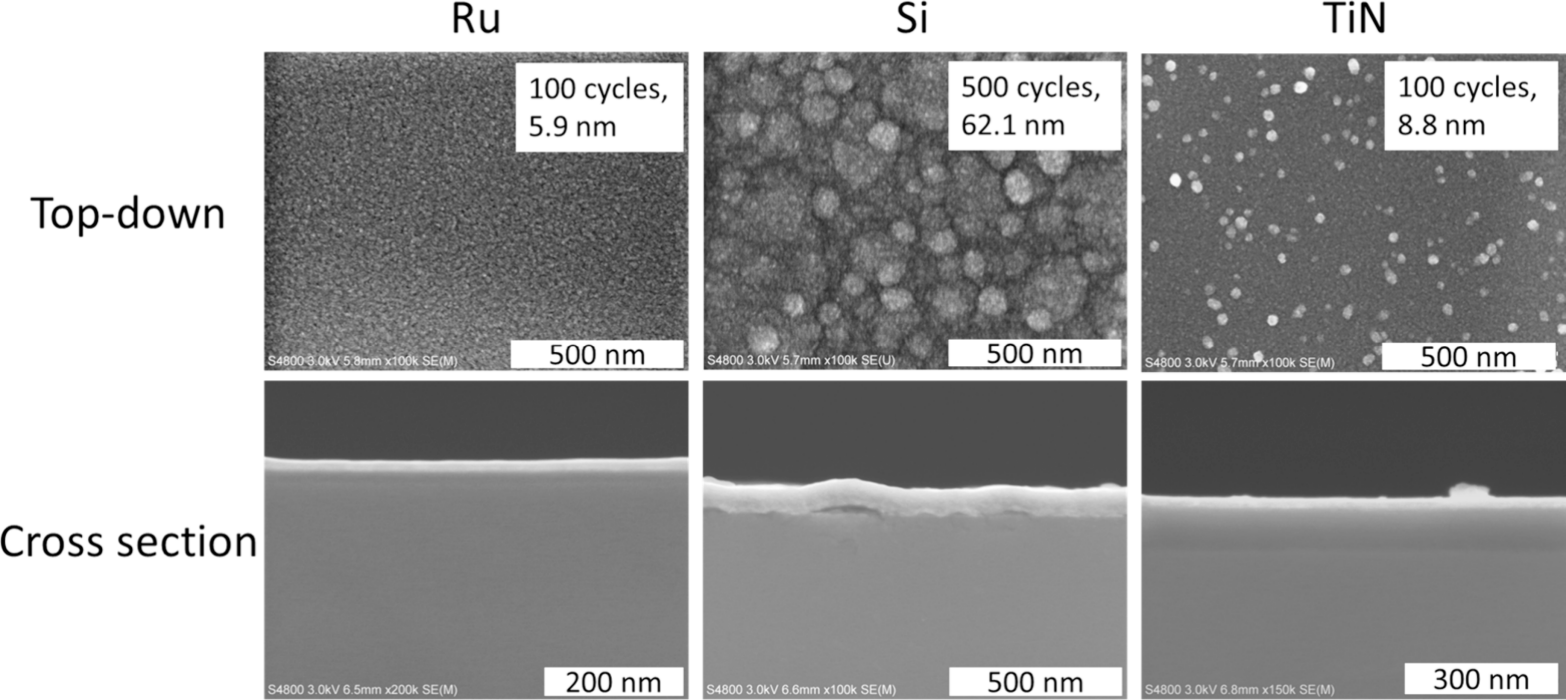
Top-down and cross-sectional SEM images of NbC_*x*_N_*y*_ films deposited on Ru, Si, and TiN substrates at 425 °C.

**Table 1:** Thermal Expansion Coefficients of Different Materials at Room Temperature

Material	CTE at RT (× 10^–6^/°C)
SiO_2_	0.26 ^52^
Si	2.6 ^53^
**NbC**	**6.2** ^54^
Ru	6.9 ^55^
TiN	9.4 ^56^

### Composition and Phase Analysis

We studied the films with ToF-ERDA, grazing incidence X-ray diffraction (GIXRD) and XPS. Figure 3b shows the atomic composition of the films measured with ToF-ERDA as a function of deposition temperature. The compositions of the films are quite constant at deposition temperatures of 250–350 °C, above which the carbon content swiftly increases (Figure 3b). Yet, the resistivities of the films decrease until 375 °C and stay within the range until 425 °C (Figure 3c). One factor that typically affects film resistivity is the ratio of carbidic carbon to amorphous carbon: the more carbidic carbon, the lower the resistivity. The increase in the carbon content above 350 °C could mostly be due to carbidic carbon being incorporated into the films as the increase in the deposition temperature provides additional thermal energy to drive the formation of Nb–C bonds. At 450 °C, the resistivity of the film increases (Figure 3c). The only observable difference between the films deposited at 425 and 450 °C is the increased oxygen content (Figure 3c). Therefore, the rise in the resistivity could be caused by the increase in the amount of oxygen being incorporated into the films (see below). On the other hand, thermogravimetric analysis (TGA) measurements (Figure 2) showed that (Me_3_Si)_2_DHP decomposes at 370 °C. However, the measurement environment does not match the deposition conditions, so it is possible that (Me_3_Si)_2_DHP decomposes at higher temperatures in our ALD reactor where its residence time is short. We cannot observe any increase in the contents of other impurity elements in the films, but at these temperatures (Me_3_Si)_2_DHP might be decomposing, and the decomposition products could incorporate amorphous carbon in the films and thereby increase the resistivity.

Figure 7a shows the ToF-ERDA elemental depth profiles of a 62 nm NbC_*x*_N_*y*_ film deposited at 425 °C. The concentrations of all the elements except for oxygen stay constant through the thickness of the film. It is apparent that the film surface has oxidized. The observed oxygen content (∼8–10 at. %) in the film bulk was an unexpected result and found at all deposition temperatures. Additionally, we observed <0.3 at. % of sodium in all the films. Since none of the precursors contain oxygen or sodium, its source might be the reactor parts. We visually observed etching of the glass and quartz parts of the reactor by NbF_5_ during the depositions. The reactions of NbF_5_ with glass at elevated temperatures can form volatile niobium oxyfluorides, which would incorporate oxygen into the films. Because glass parts are the only sodium source in the reactor, the sodium in the films is also ascribed to etching of the glass parts.

**Figure 7:**
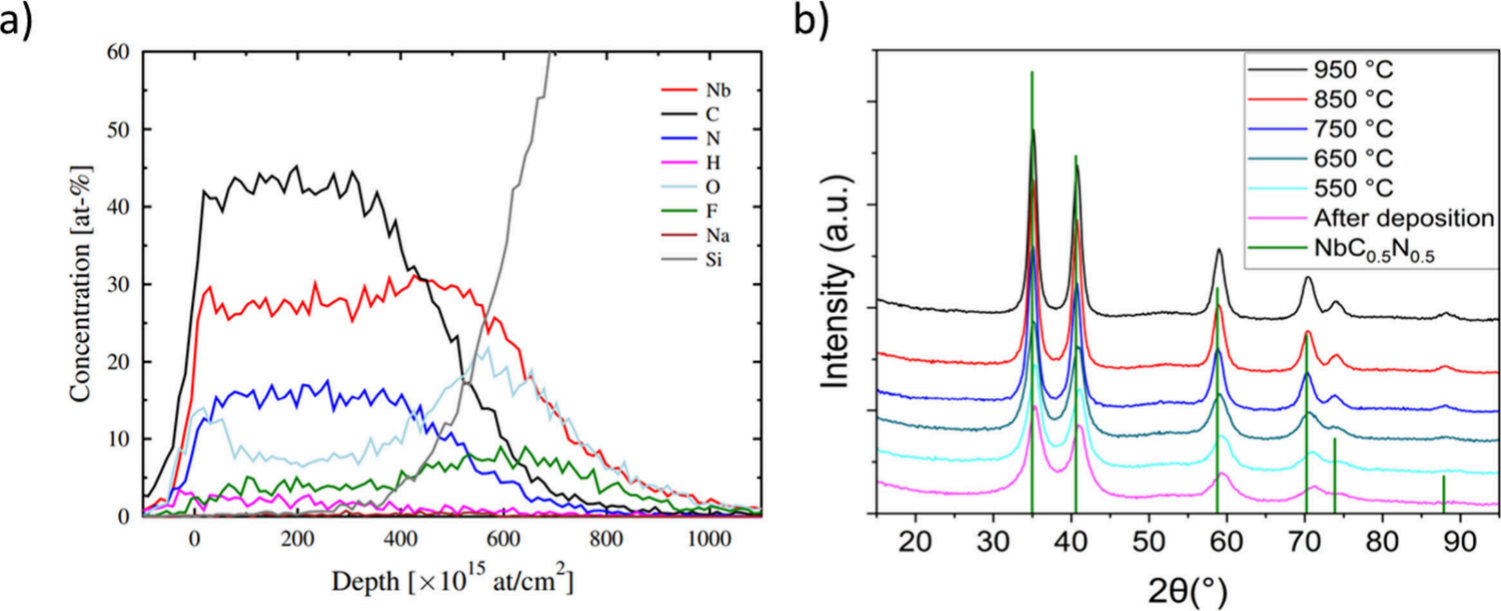
ToF-ERDA elemental depth profiles of a 62 nm NbC_*x*_N_*y*_ film deposited at 425 °C (a). GIXRD diffractograms of a film deposited at 425 °C and after annealing in a N_2_ atmosphere at different temperatures. The reference peaks correspond to cubic NbC_0.5_N_0.5_ (b).

To study the phase and crystallinity of the films, we measured GIXRD. The GIXRD of the as-deposited, ∼ 62 nm film deposited at 425 °C exhibited broad peaks (Figure 7b) which were difficult to assign to a specific phase because the reflections of cubic NbN_*x*_, NbC_*x*_, and NbC_*x*_N_*y*_ appear very close to each other. The broad peaks are likely caused by the small crystallite size of the films. After annealing the film at 550–950 °C, the peaks shifted toward lower angles as the annealing temperature increased (Figure 7b). The peaks also became narrower, indicating increasing crystallite size. The GIXRD diffractogram of the film annealed at 950 °C matched best cubic NbC_0.5_N_0.5_ (ICSD collection code: 77174, Figure 7b).

XPS provided additional information on the elemental composition and phases of the films. The XPS survey scans before and after Ar^+^ ion sputtering measured from a 62 nm film deposited at 425 °C (Figure S4) show a clear decrease of the oxygen and increase of the nitrogen content in the bulk of the film. Additionally, both the C 1s and Nb 3d peaks shift toward lower binding energies, indicating the decrease of niobium oxide and adventitious carbon contents. The N 1s spectrum of the sputtered film (Figure 8) exhibits the N–Nb peak at 397.4 eV,^57,58^ showing the nitrogen in the films to be nitridic. The C 1s spectrum shows the C–Nb peak at 282.8 eV,^59,60^ C–C and C–H components of amorphous carbon at 284.2 eV,^37,61^ and C–O at 285.5 eV.^61^ It is evident that the as-deposited films contain a large amount of amorphous carbon in addition to the carbidic carbon. The Nb 3d spectrum of the sputtered film (Figure 8) shows the Nb–C bond at 203.7 and 206.4 eV,^59,60^ and Nb–N bond at 204.0 and 206.7 eV.^57,58^ There is no indication of niobium bonding to oxygen in the bulk of the film which can be found at ∼ 203.0 (Nb 3d_5/2_) and 205.9 eV (Nb 3d_3/2_) for NbO, ∼ 205.9 (Nb 3d_5/2_) and 208.6 eV (Nb 3d_3/2_) for NbO_2_, or ∼ 207.5 (Nb 3d_5/2_) and 210.2 eV (Nb 3d_3/2_) for Nb_2_O_5_.^62^ The O 1s (Figure 8) spectrum was fitted with two peaks which were ascribed to hydroxyl species at 530.6 eV^63^ and surface −OH groups at 532.3 eV.^64^ Niobium oxides, such as Nb_2_O_5_ at 531.1 eV^65^ and NbO at 530.9 eV^65^ could not be observed.

**Figure 8:**
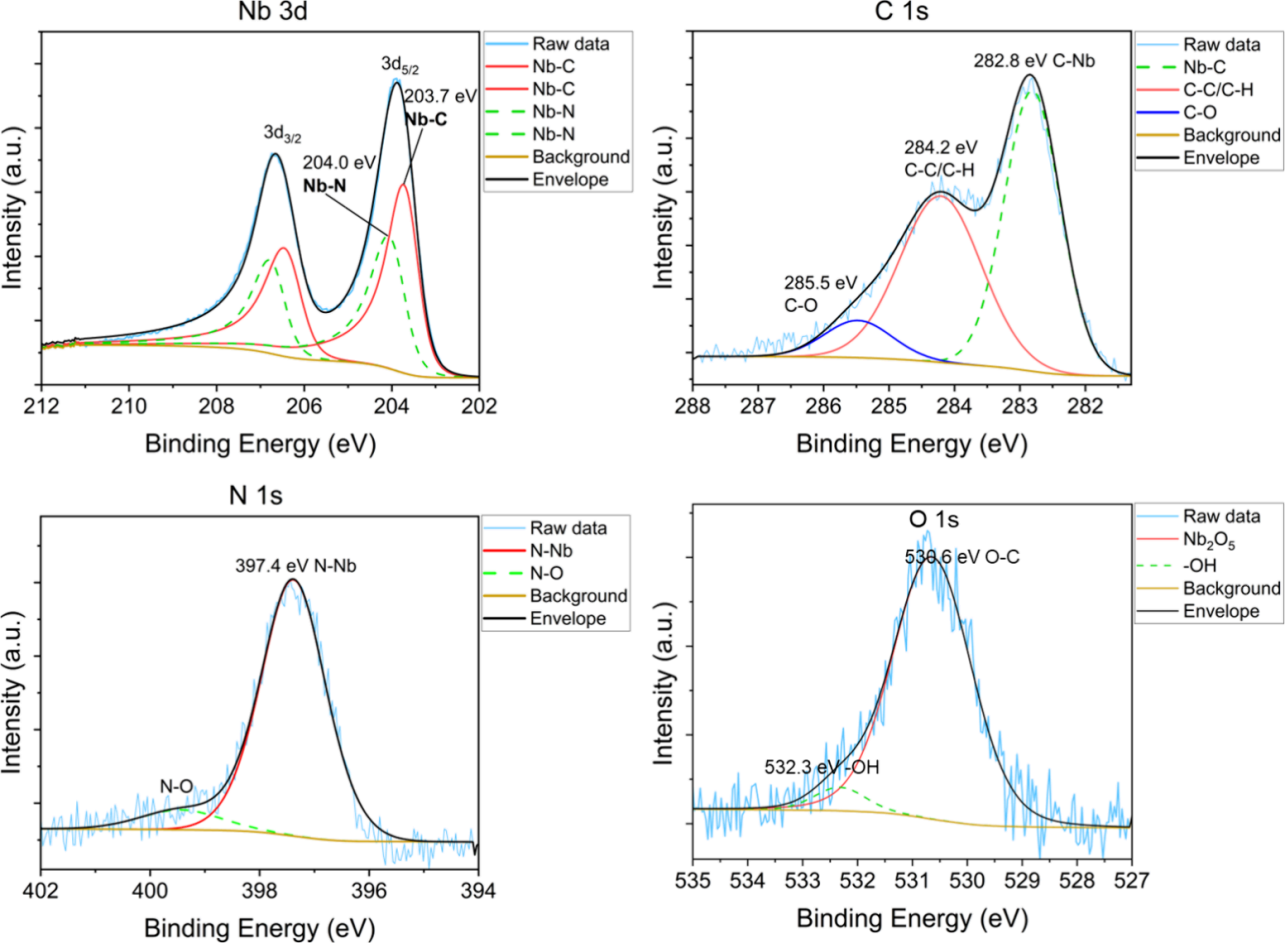
XPS Nb 3d, C 1s, and N 1s measurements of a NbC_*x*_N_*y*_ film deposited at 425 °C after Ar^+^ sputtering.

We also measured a Raman spectrum from an unannealed 62 nm film deposited at 425 °C (Figure 9). We found reference data for NbCN, NbN, and NbC to be deficient, so we are reporting our results here to provide insights on Raman analysis of NbC_*x*_N_*y*_. Very few NbCN Raman spectra have been measured, and many have been measured either only from 1200 cm^–1^ onward^66^ or the assignment of peaks is incomplete.^67^ We found the Raman modes at 126, 236, 623, 687, and 946 cm^–1^ to correspond to the cubic NbC phase.^68,69^ The presence of the characteristic D and G bands of carbon in the spectra are in agreement with XPS data, and show that some of the carbon in the films is amorphous. The first-order Raman effect of cubic NbN is forbidden, but defect-induced first-order Raman scattering with broad peaks is possible at 50–300 cm^–1^.^70^ As shown in Figure 9, it appears that we have a N-deficient cubic NbN producing the broad modes at 100–400 cm^–1^.^70^ We assigned the Raman modes at 305, 438, 521, and 946 cm^–1^ to the Si substrate, of which we have measured the spectra previously with our instrument.^71^

**Figure 9:**
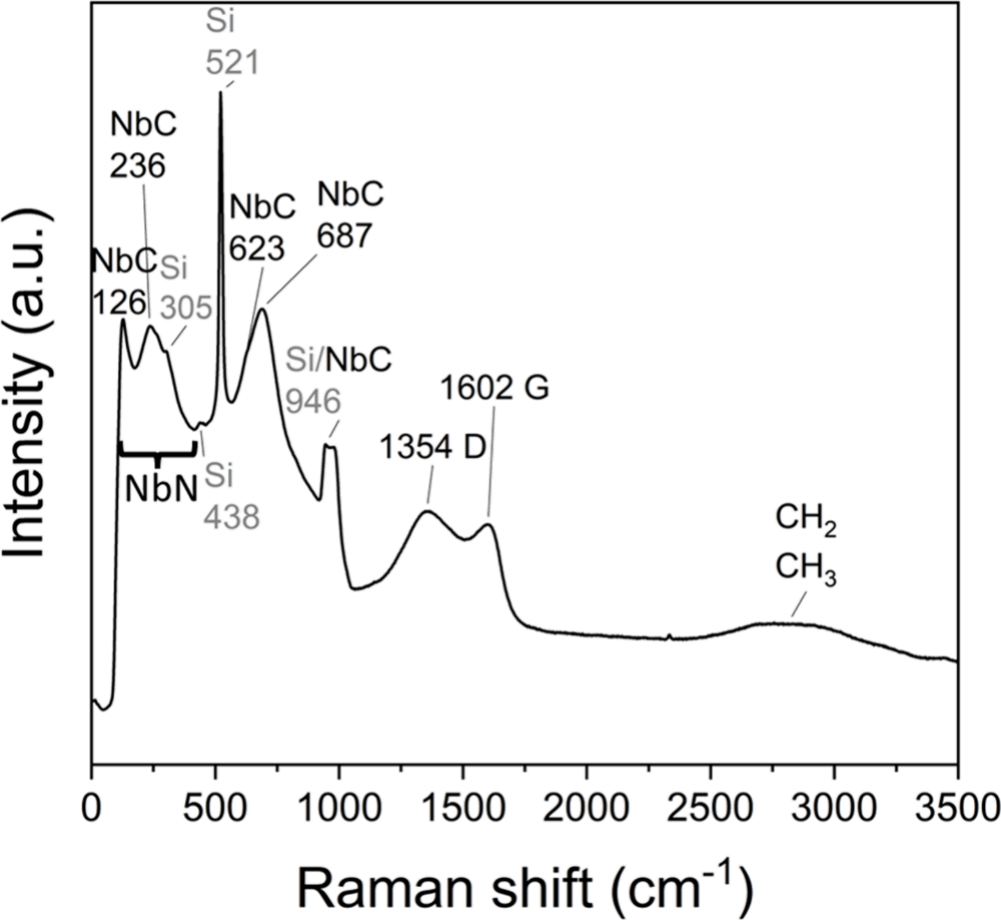
Raman spectrum of a 62 nm NbC_*x*_N_*y*_ film deposited at 425 °C.

### Annealing and Superconductivity Studies

We measured *T*_*c*_ of 62 nm thick NbC_*x*_N_*y*_ films deposited at 425 °C on Si substrates before and after annealing in N_2_ at 550, 650, 750, 850, and 950 °C (Figure 10). Additionally, a 50 nm thick film deposited at 375 °C was characterized. This film was not superconducting as-deposited at the cryostat base temperature (about 3.5 K). After annealing at 750 °C, it exhibited superconductivity below 11.0 K (Figure S5). The films deposited at 425 °C were already superconductive as-deposited with a *T*_*c*_ of 4.1 K, see Figure 10. In this figure, the as-deposited and annealed films are from different batches. However, we corroborated that the *T*_*c*_ of the as-deposited films in both batches are closely the same, see Figure S5. It is clear from Figure 10 that *T*_*c*_ steadily increases with the annealing temperature and reaches a maximum of 14.5 K after annealing at 950 °C. This is very close to the literature value for bulk NbCN (17.8 K)^6^, which is a good result for a thin film. This is also the highest *T*_*c*_ obtained for a nonoxide ALD film and second highest *T*_*c*_ obtained for any film deposited by thermal ALD. A study by Sønsteby et al.^72^ shows a *T*_*c*_ of 20 K for La_1.81_Sr_0.19_CuO_4–y_ deposited by thermal ALD. For plasma-enhanced ALD, the highest critical temperatures were observed in Nb_0.75_Ti_0.25_N annealed at 1000 °C^73^ (15.9 K) and in NbN^74–76^ films (13.8 K). The enhancing of *T*_*c*_ can be attributed to improved crystallinity as corroborated by GIXRD results in Figure 7b, as well as to lower film impurity, as will be discussed below. As expected, for as-deposited films *T*_*c*_ increases with films thickness, see Figure S6.

**Figure 10:**
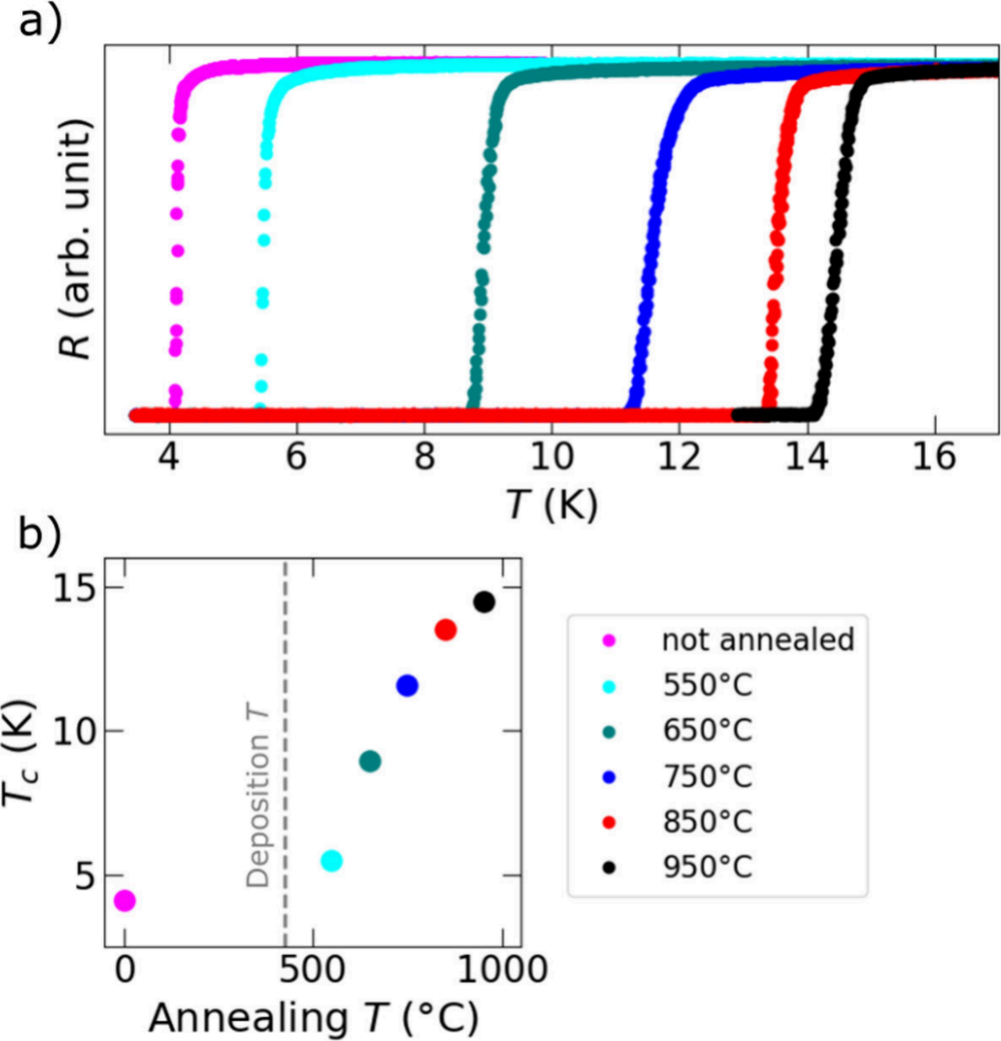
Resistance as a function of temperature for films deposited at 425 °C before annealing and after annealing at 550, 650, 750, 850, and 950 °C (a). *T*_*c*_ of the films as a function of the annealing temperature obtained from (a). The deposition temperature of 425 °C is shown with a dashed line (b).

After the annealing, we imaged the films with SEM and noticed that the films had started to peel off from the Si substrates (Figure 11). The effect is small at 550 °C, where we can see the film detaching from the substrate at the blister sites (Figure 6). Upon annealing at 750 and 950 °C, the films have rolled off the substrate at all the blister sites. The films remain intact after having rolled up. Outside the detachment blister areas, the films stayed continuous and adhered to the substrate, allowing the superconductivity measurements.

**Figure 11:**
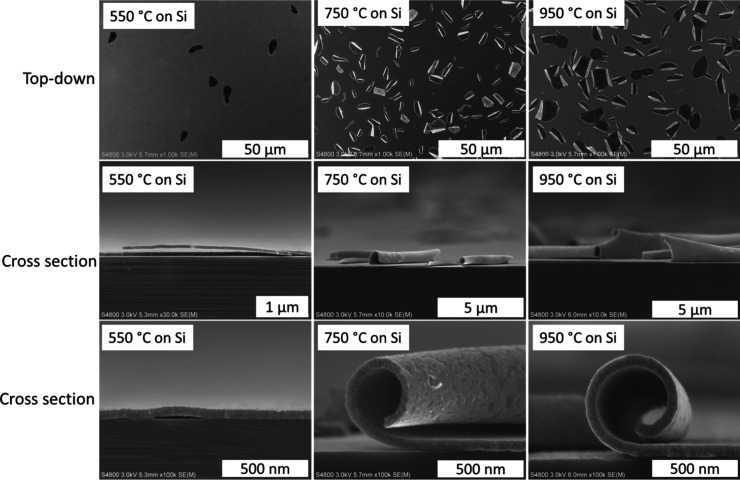
Top-down and cross-sectional SEM images of NbC_*x*_N_*y*_ films deposited on Si annealed at 550, 750, and 950 °C in N_2_.

Since the rolling off occurred only at the blister sites, it could be that the films deposited on Ru would not peel off since there are no blisters in those films. Therefore, we annealed a 17 nm NbC_*x*_N_*y*_ film deposited on Ru under N_2_ at 950 °C. SEM images taken before and after annealing can be found in Figure 12. Cavities have formed in the Si substrate, while the contrast difference between the Ru and NbC_*x*_N_*y*_ films has decreased; it looks like some of the Si has diffused into the films. XRD (Figure 12) measured from the annealed film shows that the films are no longer NbC_*x*_N_*y*_ on Ru but instead Si has reacted with both NbC_*x*_N_*y*_ and Ru, forming NbSi_2_ (COD database code: 1539276) and Ru_2_Si_3_ (COD database code: 1531861). There is no clear evidence whether Ru (COD database code: 1539052) and NbC_0.5_N_0.5_ (ICSD collection code:77174) are still present in the films since all their peaks overlap with the silicide peaks. Ru_2_Si_3_ has previously been made for example by annealing Ru thin films on Si substrates at 625 °C^77^ and NbSi_2_ by annealing slices of Nb on Si substrates at 1200 °C.^78^ Even though we can observe reactions between Si, Ru, and NbC_*x*_N_*y*_, they probably do not start below 600 °C and even then, it requires long annealing periods. We could see early stages of NbC_*x*_N_*y*_ rolling on Si already at 550 °C, so it is likely that that we would see evidence of film rolling in the annealed films on Ru if it were to happen, regardless of the reactions occurring at elevated temperatures.

**Figure 12:**
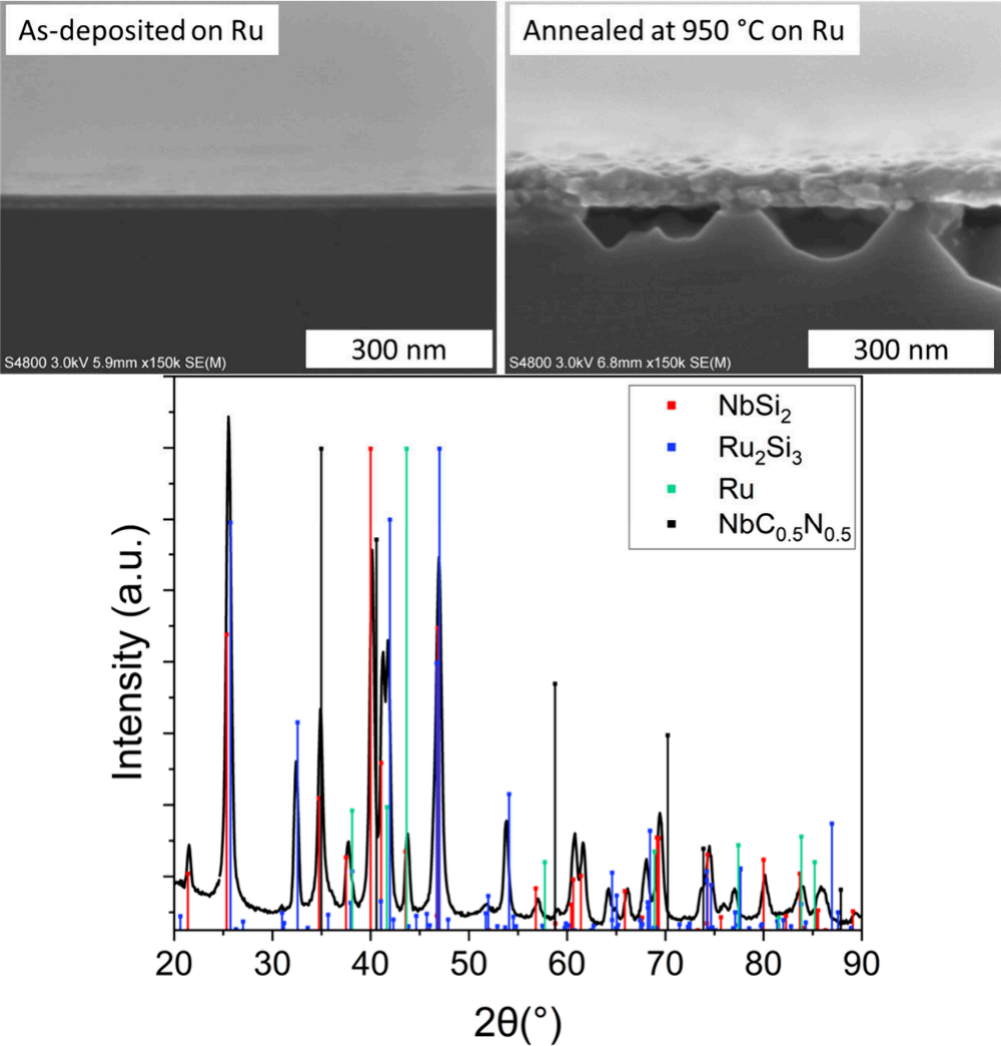
Cross sectional SEM images of a NbC_*x*_N_*y*_ film deposited on a Ru film deposited on silicon as-deposited and after annealing at 950 °C in N_2_ (top) and diffractogram of the film after the annealing (bottom).

We compared the change in the film composition by measuring ToF-ERDA from the films deposited on Si and annealed at 950 °C in N_2_ and vacuum. Table 2 shows a drastic decrease in the C content (from 42.8 to 16.5 and 20.5 at. %, respectively) after the annealing of the films. This is likely due to carbonaceous impurities cracking and desorbing as small molecules during annealing. The increase in the O content in both films is likely due to the increase of oxidized substrate–air interfaces that arise from the peeling of the films (Figure 11) and possible O residues in the vacuum annealing setup. Surprisingly, the content of nitrogen is lower for the film annealed in vacuum (Table 2) compared to the as-deposited film. To the best of our knowledge, there is only one report on the compositional analysis of NbN or NbCN films after annealing. Tian et al.^79^ studied the composition of NbN films with an electron probe microanalyzer. They compared the Nb/(N+O) ratio before and after annealing in argon at 1000 °C. They showed that the relative concentration of O did not change but the Nb/(N+O) ratio increased from ∼ 0.8 to ∼ 0.9, which indicates a loss of N atoms during annealing. Since our XPS shows that the N in our films is nitridic, which should be stable at very high temperatures, the loss of N atoms during annealing in vacuum is an interesting finding on the behavior of NbC_*x*_N_*y*_ at elevated temperatures and is subject to further studies under various annealing atmospheres. The apparent increase of the N content after annealing in N_2_ might simply be due to the decrease of the C content because the fractional increases of Nb and N are the same. However, there are multiple studies^80–82^ where NbN is fabricated by heating niobium metal to 900–1800 °C in N_2_. This indicates that also our NbC_*x*_N_*y*_ films could react with the N_2_ atmosphere. The ratio of Nb:(C,N) is 1:1 after the annealing in N_2_, which concurs with the obtained XRD result for the reference of NbC_0.5_N_0.5_ (Figure 7b), even though we have a stoichiometry of NbC_0.4_N_0.6_ in our film.

**Table 2:** ToF-ERDA Elemental Compositions of NbC_*x*_N_*y*_ Films Deposited at 425 °C before and after Annealing at 950 °C in Different Atmospheres

	[at.%]		
Element	As deposited	After annealing at 950 °C N_2_	After annealing at 950 °C vacuum
Nb	27.7	39.6	41.2
C	42.8	16.5	20.5
N	15.7	24.3	7.9
O	7.6	17.1	29.4
F	3.9	0.5	0.0
H	2.3	2.0	1.0

We observed that the *T*_*c*_ of the film annealed in vacuum at 950 °C is only 9.2 K (Figure S7), which is significantly lower than that for the films annealed in N_2_ (14.5 K). Notably, the *T*_*c*_ is still much higher than that for the as-deposited film (4.1 K), which we attribute to the improved crystallinity and lower impurity content of the annealed film. However, the film annealed under vacuum has less nitridic N than the one annealed under N_2_, which can lower the overall *T*_*c*_ since the critical temperature is lower for NbC (11.1 K)^4^ than for NbN (16.0 K)^5^ and NbCN (17.8 K)^6^. This shows that the amount of nitridic nitrogen has a substantial contribution to the *T*_*c*_ of the NbC_*x*_N_*y*_ material.

## Conclusions

We developed the first ALD process for NbC_*x*_N_*y*_ films using NbF_5_ and (Me_3_Si)_2_DHP as precursors at 250–450 °C. We deposited the films on Si, Ru, and TiN, and observed blistering of the films on Si substrates owing most likely to the difference of the thermal expansion coefficients of the substrate and the film. At 425 °C, the GPC was 1.3 Å on Si and 0.8 Å on Ru and TiN. Additionally, we annealed the films in N_2_ at 550–950 °C, after which the film was cubic NbC_0.4_N_0.6_. The film annealed at 950 °C exhibited the highest superconducting critical temperature of 14.5 K, which is the highest achieved for a nonoxide film deposited with thermal ALD.

## Supplementary Material



## Notes

Additional experimental details, methods, and figures, including measured SEM images, XRD diffractograms, XPS spectra, and *T*_c_ measurements (PDF)
