# Supplementary material for: Atomic Layer Deposition of Superconductive Niobium Carbonitride Thin Films
Source: Chem Mater. 2025 Aug 19;37(17):6770–81. doi: 10.1021/acs.chemmater.5c01456 (PMC12424125; doi:10.1021/acs.chemmater.5c01456)
Supplement: Supplementary file 1 [file cm5c01456_si_001.pdf]

Supporting Information

Atomic Layer Deposition of Superconductive Niobium Carbonitride Thin Films

Paloma Ruiz Kärkkäinen[†*], Anton Vihervaara[†], Timo Hatanpää[†], Katja Kohopää[‡], Mikko J. Heikkilä[†], Marco Marín-Suárez[‡], Kestutis Grigoras[₴], Kenichiro Mizohata[†], Georgi Popov[†], Mykhailo Chundak[†], Antti Kemppinen[‡], Matti Putkonen[†], Mikko Ritala[†*].

†Department of Chemistry, University of Helsinki, FI-00014 Helsinki, Finland

E-mail: paloma.ruizykarkkainen@helsinki.fi

mikko.ritala@helsinki.fi

‡VTT Technical Research Centre of Finland Ltd, QTF Centre of Excellence, P.O. Box 1000, FI-02044 Espoo, Finland.

₴Department of Microelectronics and Quantum Technology, VTT Technical Research Centre of Finland P.O. Box 1000, FI-02044 Espoo, Finland

Studies on the Substrate-enhanced Growth of NbC_xN_y Films on Si Substrates

To further investigate the substrate-enhanced growth behavior of NbC_xN_y on Si, we gave only one 3.0 s pulse of NbF_5 on Si at 425 °C and studied the surface with SEM, EDS, XRD, and XPS. The SEM image (Figure S1a) shows many scattered nuclei on the surface. EDS gave a nominal thickness of the initial deposit to be 0.46 nm when calculated with the density obtained for the films by XRR (4.8 g/cm^3). XRD (Figure S1b) showed two wide peaks which could not be attributed to any crystalline phase consisting of Nb, F, Si, or O.

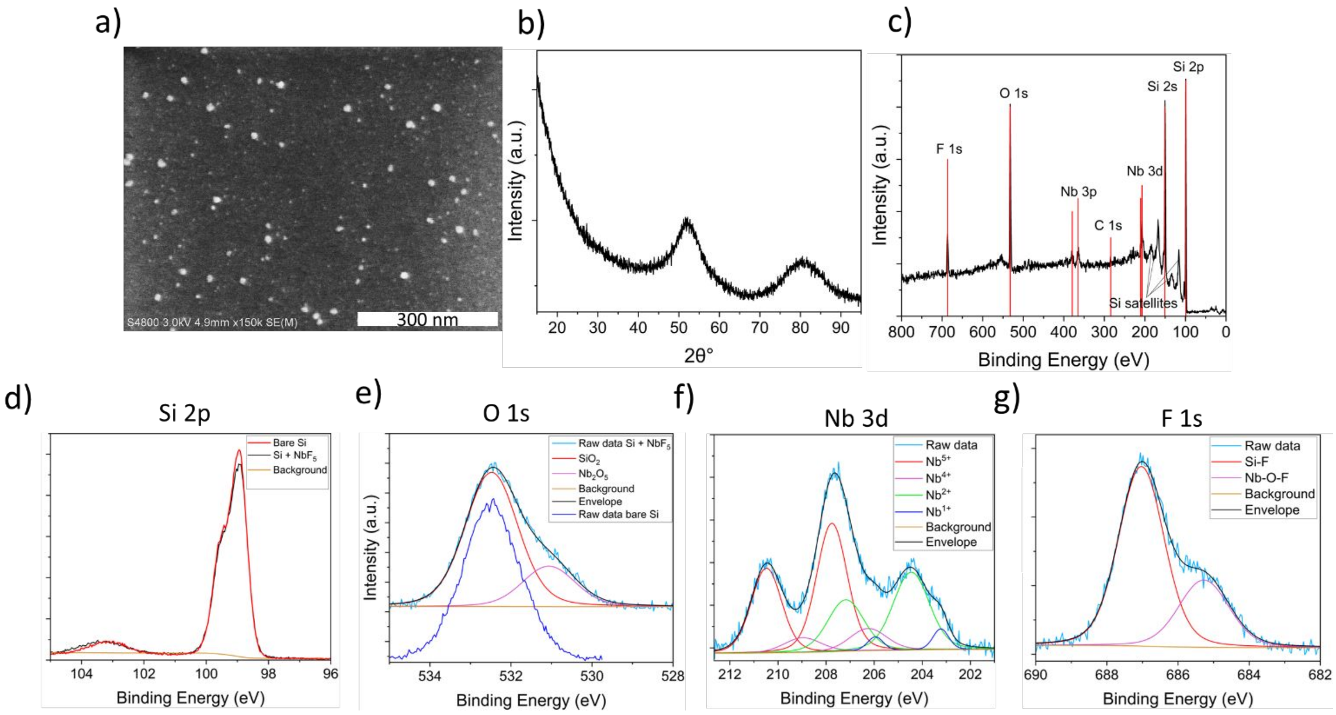

Figure S1. SEM image (a), XRD diffractogram (b), and XPS spectra (c-g) measured after giving one NbF_5 pulse on Si at 425 °C. (d) and (e) contain the spectra measured from the bare Si substrate as well.

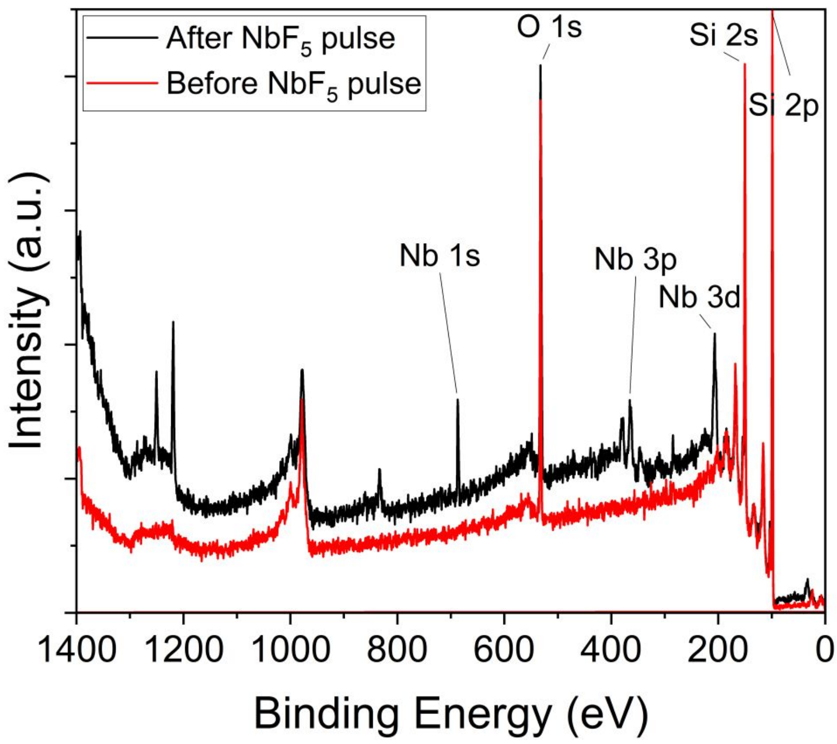

Figure S2. XPS spectra measured before and after one pulse of NbF_5 on Si substrate at 425 °C.

The sample with only one pulse of NbF_5 was transferred through ambient air from the ALD reactor to the XPS chamber. The survey XPS scans of the Si substrate with the native oxide before and after the NbF_5 pulse can be seen in Figure S2. After the NbF_5 pulse, we can already see Nb and F peaks emerge (Figure S2). A more detailed survey scan is shown in Figure S1c. The amount of carbon species was negligible. Figure S1d shows the Si 2p spectra before and after the NbF_5 pulse revealing a small shift of the peak ascribed to SiO_2 from 103.1 to 103.3 eV. We attribute this to the appearance of F and Nb species on the Si surface. The peak at ~100 eV is ascribed to Si-Si bonds[1]. The O 1s spectra measured before and after the NbF_5 pulse (Figure S1e) shows a new peak at 531.1 eV after the NbF_5 pulse, which corresponds to the presence of Nb_2O_5 (Nb^{5+}).[2] We assume that the F is distributed within the oxide layer and can be found as NbO_xF_{5-2x}. The position of the O 1s peak from the oxyfluorides overlap with the Nb^{5+} (Nb_2O_5) and Si^{4+} (SiO_2) peaks at 532.5 eV and cannot be distinguished.

We fitted the Nb 3d spectra measured after the NbF_5 pulse (Figure S1f) with four doublets and the $3d_{5/2}$ peaks were ascribed as follows: 207.7 eV as Nb^{5+}, 206.2 eV as Nb^{4+}, 204.4 eV to Nb^{2+}, and 203.2 eV to Nb^{1+}.[2] The Nb^{2+}- Nb^{5+} can be ascribed to Nb fluorides in addition to oxides arising from the transfer of the sample to the XPS chamber. We ascribe the peak at 203.2 eV to the Nb-Si bond. We fitted the F 1s spectra measured after the NbF_5 pulse (Figure

S1g) with two peaks at 685.2 eV corresponding to Nb-O-F[2], and 687.0 eV to Si-F[3]. The Si-F peak visible at 687.0 eV concurs with the peak at 203.2 eV in the Nb 3d spectra.

Supplementary Figures

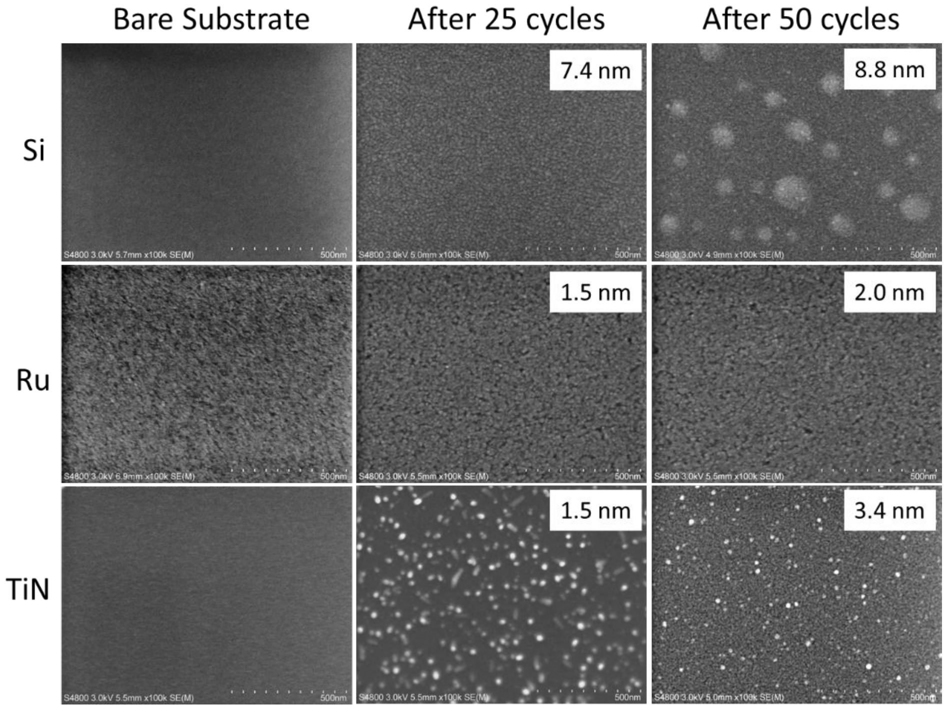

Figure S3. SEM images of NbC_xN_y films deposited at 425 °C on Si, Ru, and TiN substrates with 25 and 50 cycles.

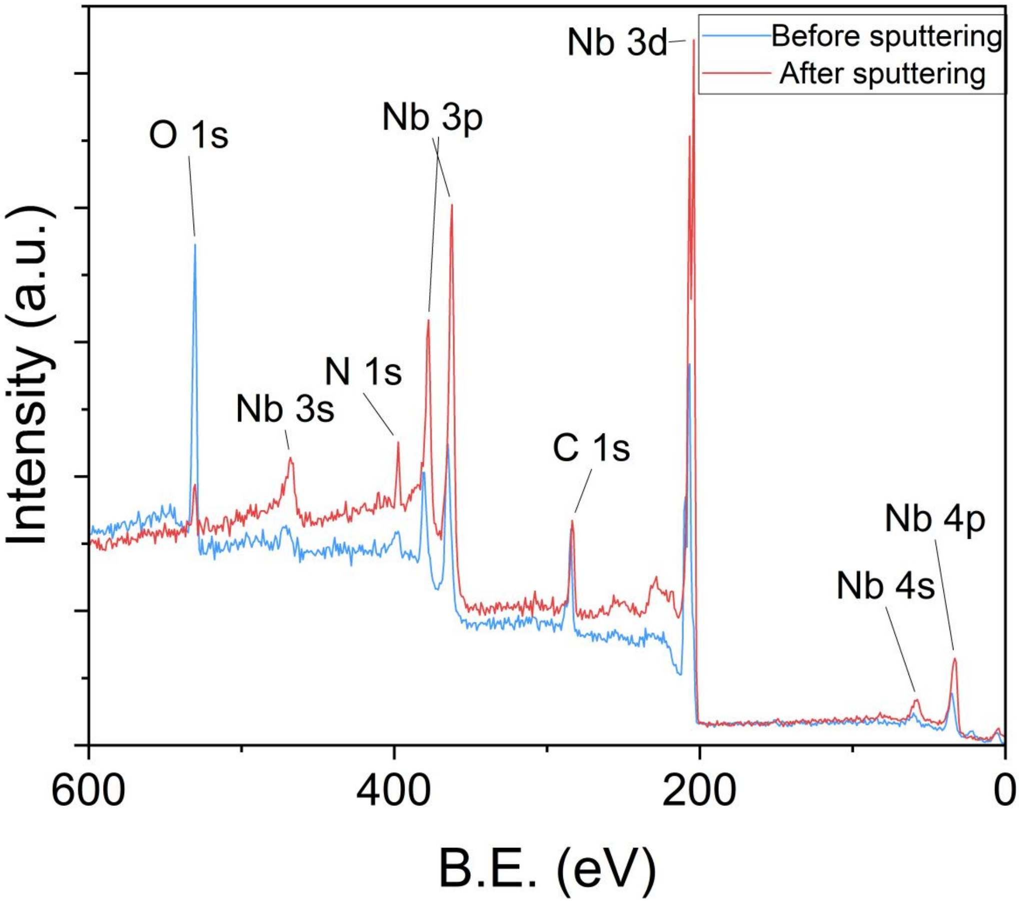

Figure S4. XPS survey scans of a 62 nm NbC_xN_y film deposited at 425 °C before and after Ar^+ sputtering.

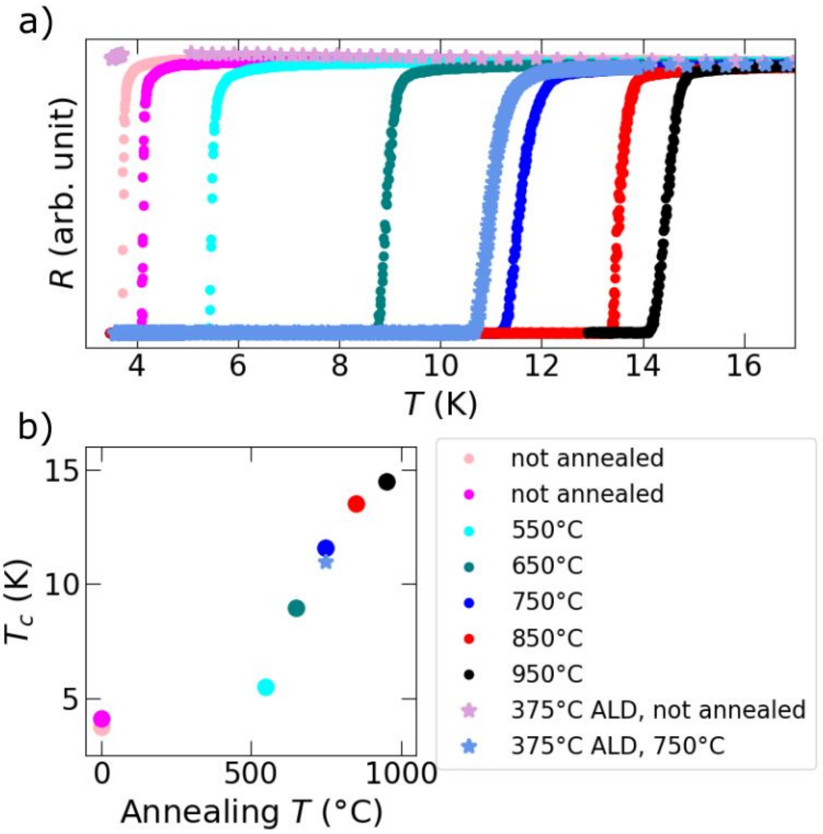

Figure S5. (a) Resistance as a function of temperature for films deposited at 425 °C before annealing and after annealing at 550, 650, 750, 850, and 950 °C and a film deposited at 375 °C before and after annealing at 750 °C. Here, we show results before annealing from two different films deposited with the same pulsing sequence at 425 °C. (b) T_c of the films as a function of the annealing temperature. We show data from Figure 9 for easier comparison and visualization.

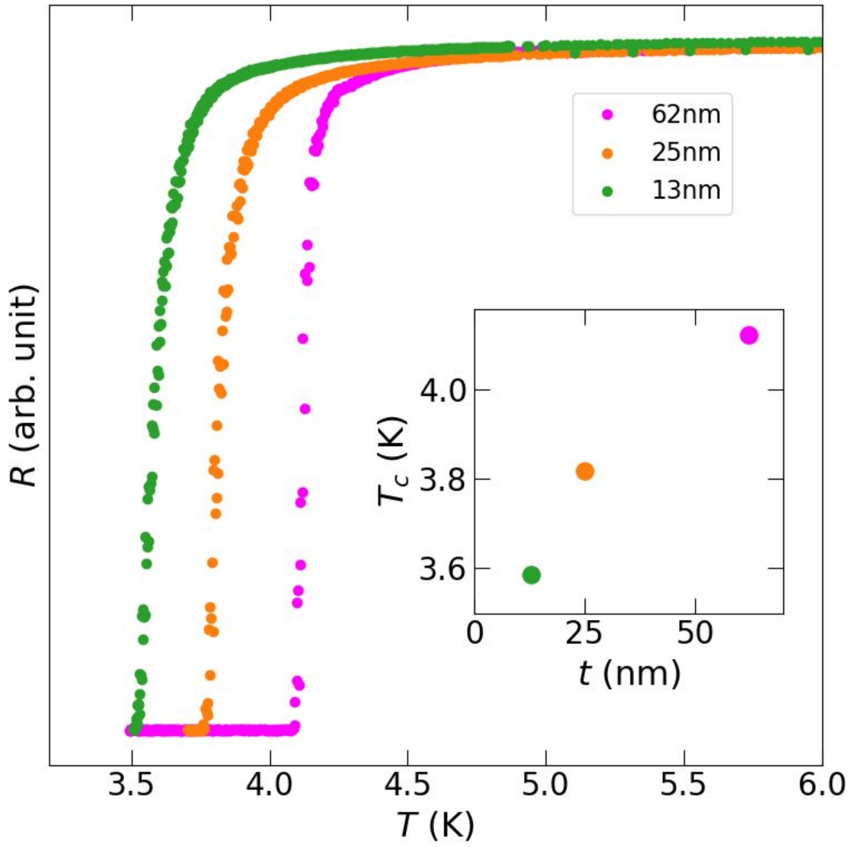

Figure S6. Resistance as a function of temperature for NbC_xN_y films of various thicknesses deposited at 425 °C. Inset: T_c as a function of film thickness.

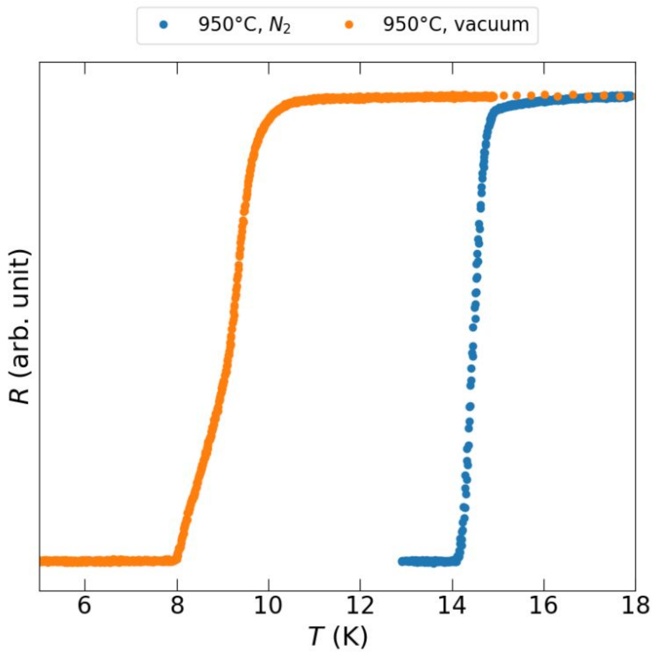

Figure S7. Resistance as a function of temperature for NbC_xN_y films deposited at 425 °C after annealing at 950 °C in N_2 and vacuum.

References

1. Taniwaki, S. *et al.* Correlation between chemical-bonding states and fixed-charge states of Sr-silicate film on Si(100) substrate. *J. Vac. Sci. Technol. A* **34**, (2016).
2. Prudnikava, A., Tamashevich, Y., Makarova, A., Smirnov, D. & Knobloch, J. In-situ synchrotron x-ray photoelectron spectroscopy study of medium-temperature baking of niobium for SRF application. *Supercond. Sci. Technol.* **37**, 075007 (2024).
3. Wen, C.-R., Frigo, S. P. & Rosenberg, R. A. Reactions of SiF4 adsorbed on Si(111)(7 × 7) at 30 K. *Surf. Sci.* **249**, 117–128 (1991).
